# Advancements in *p53*-Based Anti-Tumor Gene Therapy Research

**DOI:** 10.3390/molecules29225315

**Published:** 2024-11-11

**Authors:** Yuanwan Peng, Jinping Bai, Wang Li, Zhengding Su, Xiyao Cheng

**Affiliations:** 1 Institute of Modern Fermentation Engineering and Future Foods, School of Light Industry and Food Engineering, Guangxi University, No. 100, Daxuedong Road, Nanning 530004, China; 2216394003@st.gxu.edu.cn (Y.P.); 2316392001@st.gxu.edu.cn (J.B.); 2416393006@st.gxu.edu.cn (W.L.); 2 School of Pharmaceutical Sciences and Institute of Materia Medica, Xinjiang University, Urumqi 830017, China

**Keywords:** *p53*, gene therapy, tumor, delivery vector

## Abstract

The *p53* gene is one of the genes most closely associated with human tumors and has become a popular target for tumor drug design. Currently, *p53*-based gene therapy techniques have been developed, but these therapies face challenges such as immaturity, high safety hazards, limited efficacy, and low patient acceptance. However, researchers are no less enthusiastic about the treatment because of its theoretical potential to treat cancer. In this paper, the advances in *p53*-based gene therapy and related nucleic acid delivery technologies were reviewed and prospected in order to support further development in this field.

## 1. Introduction

The *p53* gene plays a crucial role in inhibiting tumors in humans and is the gene most closely associated with human tumors identified to date. In stress-free cells, the wild-type p53 (WTp53) protein encoded by the *WTp53* gene exists mainly in dimer form. When cells are exposed to stress signals such as hypoxia, DNA damage, and oxidative stress, the WTp53 protein is activated through phosphorylation and acetylation pathways and then assembled into tetramers. The WTp53 tetramer identifies p53 binding sites located in the promoter or enhancer of target genes through their DNA binding domain to regulate transcription, triggering cell cycle arrest [1], senescence [2,3], apoptosis [4,5], DNA repair [1], ferroptosis [6], autophagy [7,8,9], tumor angiogenesis inhibition [10,11,12], and other functions. P53 is not only a transcription factor that executes the signal but also interacts with other proteins in the cell to execute the signal. P53 in cytoplasm is also involved in regulating cellular metabolic activities, such as glycolysis [13]. In addition, p53 affects immune system function. WTp53 makes tumor cells more susceptible to immune cell attacks [14], whereas the absence of WTp53 in tumor cells limits immune system attacks on cancer cells [15].

**Figure 1:**
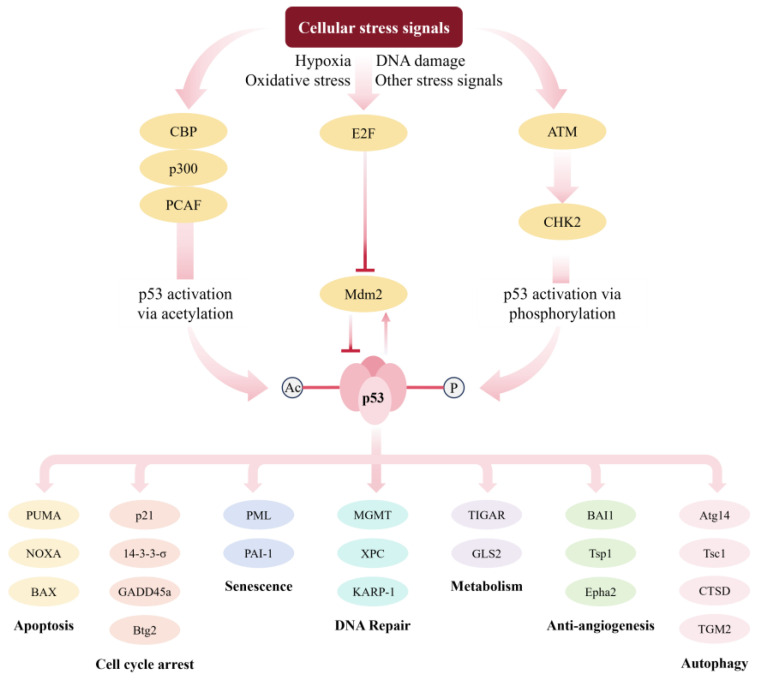
The activation and regulation of p53. Mdm2 can directly bind to p53, inhibiting its transcriptional activity. This interaction leads to the ubiquitination and proteasomal degradation of p53 as well as its export from the nucleus. The overactivity of Mdm2 may reduce the efficiency of p53 translated from a transfected vector. When cells are exposed to various stress signals, such as hypoxia, oxidative stress, or DNA damage, the pathways for p53 acetylation or phosphorylation are activated. This inhibits Mdm2-mediated ubiquitination and degradation of p53, promoting its stability and accumulation. Under pressure signals, E2F1 inhibits the expression of Mdm2, a ubiquitin ligase that promotes the degradation of p53. p300/CBP and related protein PCAF can bind and acetylate p53. The ATM activates its kinase activity, followed by phosphorylation and activation of CHEK2, which further phosphorylates the p53 protein. The phosphorylation and acetylation of p53 can inhibit its ubiquitin degradation and promote the stability and accumulation of p53. Then, p53 triggers cell apoptosis, cell cycle arrest, cell senescence, DNA repair, metabolic regulation, anti-tumor angiogenesis, and autophagy by regulating various downstream target genes or interactions with other proteins.

However, *p53* gene mutations occur in over 50% of human tumors, and the mutant *p53* (*MTp53*) gene may lose its anti-cancer activity or even become oncogenic [16,17]. Cancerous cells devoid of WTp53 are capable of enduring genomic instability and amplified cancer-causing signals, and the advancement of cancer is markedly hastened when the *p53* gene is missing or altered [18]. Even when tumor cells carry WTp53, the anti-cancer activity of WTp53 may be reduced due to the overexpression of Mdm2 and MdmX [19,20]. Given the strong link between p53 and the progression of tumors (illustrated in Figure 1), targeting p53 has emerged as a popular approach in formulating anti-cancer therapies.

Gene therapy’s fundamental concept involves moving standard or other therapeutic external genes to specific cells to make up for faulty genes, mute detrimental genes, or rectify abnormal genes [21]. Cancer is caused by genetic mutation; therefore, gene therapy has long been considered a radical cure for cancer, theoretically demonstrating great potential to fight cancer. Gene therapy has many advantages. First, conventional drug design methods are difficult to implement due to the lack of binding pockets or conformational sites for p53 proteins. P53 is often considered an untreatable target, but luckily, gene therapy can overcome this. Second, gene therapy drugs are nucleic acid drugs, including DNA and RNA. Nucleic acid drugs can be produced quickly and on a large scale compared to conventional small molecule or protein drugs. The development of protein drugs requires a process from gene construction to protein expression and purification. High-purity proteins are often difficult to obtain, and the purification process is complex; in addition, gene therapy drugs do not require in vitro expression and purification. As a result, nucleic acid drugs have a relatively short development cycle. Third, gene therapy usually has more long-lasting efficacy, which can reduce the frequency of administration and lower the medication burden for patients.

Currently, a range of *p53*-based gene therapies have been established, yet to date, there has not been a discovery of a drug in the field that is both highly effective and broadly acknowledged. This could be linked to issues like flaws in gene therapy techniques and underdeveloped nucleic acid delivery systems. Due to the theoretically significant potential of this therapy in treating tumors, researchers’ enthusiasm for this therapy has never diminished. Overcoming these current limitations could enable *p53*-based gene therapy to fully leverage its benefits and potentially contribute significantly to cancer therapy.

## 2. Varieties of *p53*-Based Gene Therapy Techniques

**Figure 2:**
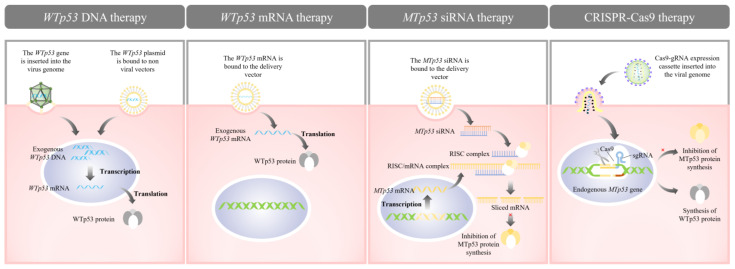
Varieties of *p53*-based gene therapies.

The fundamental tenets of *p53*-based gene therapy primarily involve elevating WTp53 levels in cancerous cells and diminishing MTp53 amounts in these cells. Presently, the established therapeutic approaches include *WTp53* DNA replacement therapy, *WTp53* mRNA replacement therapy, and small interfering RNA (siRNA) therapy directed towards *MTp53*, along with others (illustrated in Figure 2). Notably, *WTp53* DNA replacement therapy has been extensively and thoroughly researched, whereas most other therapies are still in the nascent stages of exploration and have yet to be translated into clinical practice.

### 2.1. WTp53 DNA Therapy

*WTp53* DNA therapy, also known as *p53* gene therapy, involves inserting *WTp53* DNA into cancer cells and producing excessive WTp53 protein, causing cell apoptosis or inhibiting cancer cell growth. This is primarily accomplished by integrating the *WTp53* expression cassette into the virus genome and infecting cells with the virus or by supplying *WTp53* plasmid DNA (pDNA) to cells through non-viral vectors. Scientists have carried out a great deal of basic and clinical research, achieved some results in tumor treatment, and accumulated valuable experience for future development. Table 1 describes the progress of various *p53* gene therapies. Some researchers have proposed directly introducing p53 protein into tumor cells [22,23], but this approach faces challenges due to the short half-life of protein molecules and the complexities involved in their purification. Additionally, in comparison to gene therapy, this method struggles to achieve long-lasting therapeutic effects. Consequently, gene therapy continues to be a more advantageous choice.

This treatment based on exogenous DNA overexpression is a rapidly evolving gene therapy. In 2015, a genetically modified herpesvirus therapy for treating melanoma was approved for market, named T-Vec, which was the first oncolytic therapy or gene therapy approved in the United States [51]. T-Vec can replicate and synthesize granulocyte macrophage colony-stimulating factor in tumor cells, leading to the lysis and release of tumor antigens, thereby triggering an immune response to kill metastatic melanoma cells. In 2017, Luxturna received FDA approval, reportedly the first direct administered gene therapy approved in the United States [52]. Luxturna is a drug that uses viral vectors to deliver genes for human retinal pigment-specific proteins to patients with retinal dystrophy associated with gene mutations, promoting the expression of all trans retinal ester isomerases in patients and restoring vision [52]. While *p53* DNA therapy is well documented in basic and clinical studies, there are no FDA-approved drugs on the market. In clinical settings, the *p53* gene therapy frequently proves inadequate for enhancing patient survival rates [53]. Therefore, how to further improve and innovate this treatment to overcome the current predicament is a question we must ponder.

Researchers are attempting to improve the efficiency of therapies by optimizing plasmids. The supercoiled pDNA are believed to be easier to achieve high-level protein expression than natural pDNA [54]. Higher levels of p53 protein expression were obtained by researchers transfecting tumor cells with pure supercoiled *p53* pDNA [54]. But this may increase purification costs. Recently, a *p53* microcycle DNA (mcDNA) is also being developed for gene therapy [55]. McDNA is a novel small loop super helix expression box obtained through site-specific recombination of traditional pDNA in bacteria. McDNA, unlike pDNA, lacks bacterial elements like resistance marker genes and replication origins, enhancing its safety [56]. In addition, the sequence of mcDNA is shorter and has been shown to be easier to transduce and achieve long-term effective protein expression [55]. However, mcDNA currently faces difficulties in purification processes and economic costs.

The limited global acceptance of *WTp53* replacement therapy can be attributed to its minimal tumor-fighting impact in isolation. Researchers frequently integrate *WTp53* replacement therapy with chemotherapy, radiotherapy, immunotherapy, and various other cancer treatments to enhance their anti-cancer impact. WTp53 not only induces tumor cell cycle arrest and apoptosis but also enhances the sensitivity of tumor cells to other anti-tumor drugs. Studies have shown that WTp53 represses the expression of multidrug resistance 1 (*MDR-1*) genes [57], certain MDR-associated protein (*MRP*) genes [58], and breast cancer resistance protein (*BCRP*) genes [59] in cells. Conversely, some members of the ABC transporter family, such as MRP-1, are upregulated due to MTp53 [58]. Consequently, merging *p53* gene therapy with various other treatments frequently results in synergistic outcomes. The emergence of resistance to multiple drugs in cancerous cells poses a significant barrier to cancer therapy, resulting in numerous anti-cancer medications or treatments struggling to achieve their desired outcomes. Consequently, integrating *p53* gene therapy with various other treatments holds considerable importance.

Recombinant human p53 adenovirus (rAd-p53) in combination with chemotherapy is common. *p53* gene therapy not only increases tumor cells’ sensitivity to chemotherapy but also significantly enhances anti-tumor activity while reducing the dose of chemotherapeutic agents, thereby reducing the toxicity of high-dose chemotherapy agents to humans. For example, Priya et al. [60] used a combination of the *WTp53* gene and trace doxorubicin (Dox) (1 μM) to mediate up to 98% of tumor cell mortality rate, whereas treatment with the same amount of *p53* genes and higher levels of Dox (3.13 μM) alone resulted in only 59% and 50% tumor cell mortality rate. *p53* gene therapy may play a similar role in radiotherapy [61]. Many clinical reports have shown that the combination of *p53* gene therapy with chemotherapy or radiotherapy can effectively control disease progression, improve progression-free survival time in cancer patients, and lead to better outcomes than gene therapy or radiotherapy [62,63]. However, while radiotherapy and some chemotherapy medications fight cancer through DNA damage in cancer cells, WTp53 is capable of reacting to DNA damage indicators to initiate the DNA repair process. Consequently, our worry lies in the potential of *p53* gene therapy to negate the cancer-fighting impacts of radiotherapy and chemotherapy.

Recently, the combination strategy of *p53* gene therapy and immunotherapy has been extensively explored, with promising results in the laboratory stage. Qiao et al. [64] demonstrated that the combination of interleukin-2 and rAd-p53 increased cell apoptosis levels, caused tumor regression, and prolonged survival in tumor-bearing mice. Interleukin-2 is an anti-cancer cytokine that stimulates T cell proliferation and induces innate and adaptive immunity [64]. Immune checkpoint inhibitors are immunotherapies that enhance the body’s ability to attack cancer cells, but most cancer patients do not respond to or develop resistance to such treatments [65]. Recently, research has shown that *p53* gene therapy can reverse the resistance of tumor cells to immune checkpoint inhibitors [65].

The combination of *p53* gene therapy with surgical resection and thermal therapy also demonstrated better clinical outcomes than monotherapy. Many clinical reports have shown that tumor resection surgery combined with *p53* gene therapy can reduce the postoperative recurrence or metastasis rate, improve the surgical cure rate, and improve the progression free survival and overall survival of tumor patients [21,66,67], but the relevant mechanisms are not clear. In addition, research has found that high temperatures can inhibit cancer progression by activating the transcription of *p53*. After receiving hyperthermia, the transcription activity of *p53* is significantly increased in cells [68]. Clinical studies have shown that the combination of rAd-p53 injection and local hyperthermia and chemotherapy can achieve high disease control rates and pain relief rates in cancer treatment [69].

Clinically, scientists have explored the integration of *p53* gene therapy into conventional Chinese medical practices. During clinical tests, the traditional Chinese remedy Wuling San, along with rAd-p53 injection, was employed to address malignant pericardial effusion [70]. The results showed that compared with cisplatin treatment, Wuling San combined with rAd-p53 had better clinical efficacy (*p* < 0.05) and lower incidence of adverse reactions (*p* < 0.05) [70].

Strategies were also proposed to combine *p53* with anti-tumor agents such as curcumin [53,71], lenvatinib [71], and bortezomib [17]. In addition, in basic studies, multigene therapy strategies such as *p53* gene therapy in combination with siRNA oncology therapy [72] and *p53* gene therapy in combination with gene suicide therapy [73,74] have evolved. These combination strategies have shown promising anti-tumor effects both in vitro and in vivo.

Combining *WTp53* replacement therapy with other therapies is important to synergize anti-tumor efficacy and reduce the dose-toxicity of anticancer drugs such as radiotherapy. However, it is worth noting that when combining multiple drugs, careful consideration should be given to the toxic side effects of drug interactions to ensure the safety of cancer treatment. Additionally, the limited anti-cancer effect of *WTp53* alone should not be overlooked, as this could undermine the therapy’s overall advantage compared to other anti-tumor drugs.

### 2.2. WTp53 mRNA Therapy

The process of *WTp53* mRNA therapy involves introducing *WTp53* mRNA to boost WTp53 protein levels in cancer cells, thus either suppressing or eradicating tumors. The preliminary development of *p53* mRNA therapy has recently demonstrated effective inhibition of tumor cells in lab studies [75,76]. Compared to DNA, mRNA does not need to enter the nucleus to reach the cytoplasm, making drug delivery in gene therapy less difficult. The integration of mRNA into the host genome is unnecessary, thereby eliminating the likelihood of insertion mutations. A significant number of mRNAs undergo natural degradation post-translation, thus maintaining a degree of safety [77]. Nonetheless, as this treatment is in its initial phase and devoid of practical clinical experience, rectifying its present limitations might require an extended period to more effectively transition into clinical settings.

This technology, which is based on in vitro mRNA to guide protein synthesis in vivo, has been widely applied in the field of virus prevention. Amidst the COVID-19 pandemic, the inaugural mRNA novel coronavirus pneumonia vaccine received emergency usage authorization, and the FDA sanctioned its marketing, demonstrating remarkable safety and protective effectiveness across numerous clinical records. In addition, mRNA preventive vaccines related to influenza, rabies virus, cytomegalovirus, respiratory syncytial virus, etc., have also accumulated a wealth of clinical research experience. All of these have injected tremendous confidence and development momentum into *WTp53* mRNA therapy. In these related areas, researchers improved the performance of mRNA technology products by optimizing mRNA sequences [78], chemically modifying mRNA [79], and improving mRNA purification processes [80]. These methods could contribute to the development of *WTp53* mRNA therapy. However, we should also be aware that sequence optimization should not lead to the production of unwanted or harmful proteins in cells, nor should it affect the accuracy of structural folding of WTp53 proteins.

### 2.3. MTp53 siRNA Therapy

While the previously described techniques for enhancing WTp53 expression yield some outcomes, the presence of MTp53 in tumors could disrupt *WTp53* replacement therapy, potentially resulting in unfavorable treatment results and prognoses [81]. MTp53 may bind to WTp53 to produce heterotetramers, thereby hindering the function of WTp53 [82]. In the mutation types of *p53* gene, missense mutations account for about 90%, and most mutations are located in DNA binding domain [83,84]. Therefore, many MTp53 proteins lose their ability to bind to DNA and regulate transcription, thereby losing a series of anti-cancer functions. In addition, many MTp53 proteins also acquire new carcinogenic activity to promote cancer progression. P53^R175H^ and p53^R273H^ are common missense mutation types that are more prone to intracellular aggregation than WTp53, and their pathological aggregation can lead to various cancers [85]. P53^R248Q^ is also a common missense mutation that can sustainably activate the STAT3 signaling pathway, thereby promoting cancer cell migration [86]. In addition, MTp53 protein can also promote tumor cell proliferation, genomic instability [87], and cancer resistance to chemotherapy [88]. Consequently, the possible adverse effects of MTp53 on cancer sufferers must not be overlooked.

SiRNA is capable of muting mRNA produced by specific genes and preventing the synthesis of those proteins. siRNA-based therapies do not require interference with DNA and do not permanently modify the genome, so the toxic side effects are relatively small. The FDA sanctioned Patisiran, the inaugural siRNA-based medication for transthyretin-induced cardiac amyloidosis, in 2018, proving its effectiveness and safety. Subsequently, the FDA approved more siRNA-based drugs, including Givlaari, Oxlumo, Leqvio, and Amvuttra. In cancer treatment, many siRNA-based drugs are undergoing clinical trials. The triumph of these associated medications offers guidance and confidence for researchers in developing siRNA treatments aimed at *MTp53*.

There has been consistent advocacy for creating siRNA therapies aimed at the *MTp53* gene to eradicate MTp53 in cancer cells [89]. Studies indicate that reducing MTp53 levels in cancer cells can markedly increase the rate of tumor cell death and suppression [90]. Recently, Ubby et al. [91] designed a series of *MTp53* siRNAs that can highly specifically silence multiple *MTp53* (R175H, R248W, R249S, and R273H) without affecting *WTp53* and demonstrate the ability to inhibit tumor cells. However, it is difficult to assess whether siRNA therapy affects the normal expression of other genes in the body and whether there is potential harm. Therefore, it is necessary to pay attention to the improvement of siRNA specificity and the detection methods for non-specific binding.

### 2.4. CRISPR-Cas9 Therapy

CRISPR-Cas9 is a commonly used gene editing tool based on the idea that the Cas9 protein forms a ribonucleoprotein complex with single guide RNA (sgRNA), cutting off target sites to form DNA double-strand breaks. Subsequently, the host cells were repaired mainly by non-homologous end joining and homology directed repair [92]. The non-homologous end joining pathway connects directly to the severed end, leading to mutations such as insertion or deletion, while the homology-directed repair pathway relies on homologous repair templates for precise repair or insertion to achieve genomic modifications such as knockout and base editing [92]. CRISPR-Cas9 is currently being gradually used for disease treatment. In 2019, researchers transplanted CRISPR-edited hematopoietic stem cells and progenitor cells into patients with AIDS and leukemia, alleviating acute lymphoblastic leukemia in patients, and initially explored the safety and feasibility of gene editing in clinical applications [93]. In 2022, the gene-editing drug VERVE-101 was approved for clinical trials in New Zealand, becoming the first clinical project to directly edit genes in vivo. The drug aims to treat heterozygous familial hypercholesterolemia by inhibiting the *PCSK9* pathogenic gene [94]. Recently, an in vivo CRISPR gene editing therapy called NTLA-2002, which targets the knockout of the *KLKB1* gene to prevent hereditary angioedema, showed some efficacy in clinical trials [95].

Given the CRISPR-Cas9 system’s capacity to modify the genome and rectify random mutations, there is a suggestion for a tumor therapy approach employing CRISPR-Cas9 to amend inherent *MTp53* [96]. Recently, Jordi et al. [97] successfully used CRISPR-Cas9 technology to knock out the expression of p53 protein in human lung adenocarcinoma cells in vitro, and planned to add a normal copy of *p53* to the same gene delivery system to correct abnormal genes. This study opens up new possibilities for cancer treatment.

However, there are still many problems with gene editing technology, and CRISPR-Cas9 often leads to chromosomes being lost [98], displaced, or broken [99,100], with serious safety risks. Severe adverse reactions to VERVE-101, including one death, were found in clinical trials [94]. Based on CRISPR-Cas9’s proven significant off-target effects, scientists are actively pursuing a range of strategies to reduce off-target effects, including titration doses [101], the optimization of sgRNA [102], and the modification or mutation of Cas9 [103] to improve gene editing systems. In addition, scientists upgraded algorithm programs that predict potential off-target effects to assist people and prevent off-target effects [104]. These provide guidance for the application of CRISPR-Cas9 in anti-tumor applications.

### 2.5. Therapies Involving microRNAs Related to p53

MicroRNAs (miRNAs) are a type of single-stranded RNA molecule, approximately 22 nucleotides long, that are encoded by endogenous genes [105]. MiRNAs can inhibit the translation of target mRNAs or promote their degradation, leading to changes in the levels of specific proteins. MiR-34a is a miRNA closely associated with p53. The activation of WTp53 can upregulate the expression of miR-34a, which in turn can activate WTp53 by targeting its inhibitors [106]. MiR-34a can exert tumor-suppressive effects by inhibiting cell proliferation, promoting apoptosis, and regulating tumor angiogenesis and metastasis. This suggests that miR-34a may be an effective tool for cancer treatment [106].

The 2024 Nobel Prize in Physiology or Medicine has been awarded to scientists for their discovery of miRNA and its role in post-transcriptional gene regulation. MiRNA therapy is poised to become a significant area of research in the future. Currently, some studies are focusing on miR-34a to explore its potential clinical applications. The miR-34a replacement strategy has been studied in clinical trials; however, the results have not been satisfactory. This may be due to the poor stability of miR-34a or the occurrence of severe immune responses [107]. Recently, Abdelaal et al. [108,109] developed various ligand-modified miR-34a molecules for tumor suppression, all of which demonstrated promising results and significant potential for further development. The modification strategies employed for these miRNAs are crucial for advancing their clinical applications.

## 3. Techniques for Delivering Nucleic Acids in *p53*-Based Gene Therapy

**Figure 3:**
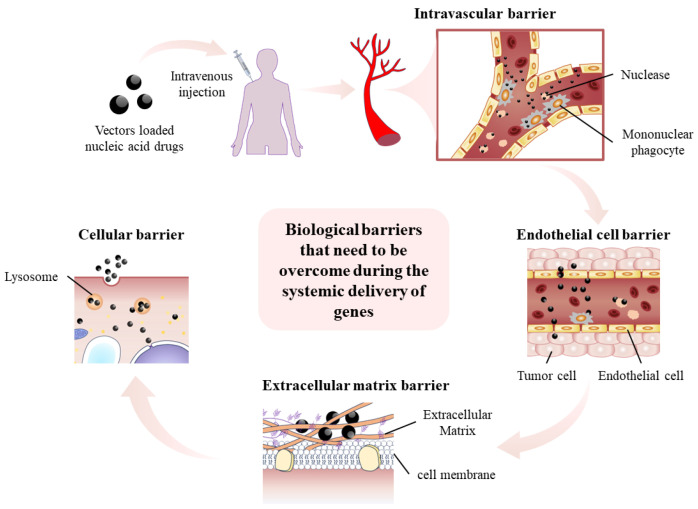
Overcoming biological obstacles in the process of nucleic acid transport.

In *p53*-based gene therapy, it is vital to precisely transport the nucleic acid intended for treatment to either the nucleus or cytoplasm in cancer cells. Nonetheless, when delivering nucleic acids, numerous biological obstacles arise, making the employment of gene delivery vectors unavoidable. Gene vectors can assist nucleic acid in overcoming biological barriers in the human body, such as nucleases in serum, mononuclear phagocytic cell system, endothelial cells, extracellular matrix, cell membrane, lysosomes, etc., making nucleic acid more effectively delivered to a target location (illustrated in Figure 3). Gene vectors are mainly divided into two categories: viral vectors and non-viral vectors. Next, we will introduce and discuss the *p53*-based gene therapy mediated by these vectors.

### 3.1. Virus Vector Mediated p53-Based Gene Therapy

The majority of viral vectors naturally bind to human cells and are adept at moving external genes to the cytoplasm or nucleus of the target cells, rendering them ideal for gene therapy. Currently, viral carriers like retrovirus, adenovirus, vaccinia viruses, and phage vectors are used in both in vivo and in vitro experiments for *p53* gene therapy. Due to safety considerations, gene therapy virus vectors tend to be those with replication deficiencies or conditional replication that have been subject to detoxification modifications. However, the long production cycle of viral gene therapy products, their susceptibility to inactivation at room temperature, and the stringent storage and transport conditions make gene therapy more expensive.

#### 3.1.1. Retrovirus Vectors

Retroviruses can efficiently deliver exogenous genes to host cells and integrate them into the host genome, ensuring their sustained and stable expression. They are often used as gene delivery vectors in early studies of *p53* gene therapy in vitro [110]. However, the integration of the retroviral genome has potential carcinogenicity for the body. Strong promoter and enhancer elements in the long terminal repeat region of some retroviruses can activate genes near the integration site through the insertion of mutagenesis, which may lead to the inactivation of tumor suppressor genes or the activation of oncogenes, thereby triggering cancer [111]. Many serious adverse events of acute leukemia in patients caused by retroviral insertion mutagenesis have been found in clinical practice [112,113,114]. Later, people made a series of improvements to retroviral vectors, such as deleting the promoter or enhancer of long terminal repeat region [115] or using blocking elements to isolate the impact of transgenic promoters and enhancers on genes near the integration site [116]. In addition, lentiviral vectors based on HIV are considered to have a lower risk of carcinogenesis due to their integration sites located within transcriptional units [117]. So far, many clinical trials have been conducted on lentivirus-related products, and no reports of human carcinogenesis have been found. This provides a new approach for *p53* gene therapy, but the safety of lentivirus still needs more validation of its usage results [117].

#### 3.1.2. Adenovirus Vector

Adenovirus vectors have been widely used in clinical settings. It does not integrate the viral genome into the host genome, avoiding the cancer risks associated with insertion of mutations, and is relatively safe for humans. However, in contrast to retroviral vectors, gene therapy mediated by adenovirus vectors exhibits comparatively brief effects and might necessitate multiple administrations. The types of rAd-p53 products mainly include replication-deficient p53 adenovirus (RDAd-p53) and conditional replication p53 adenovirus (CRAd-p53).

The construction of RDAd-p53 is generally achieved by deleting the *E1* gene of adenovirus, which is composed of *E1A* and *E1B* and encodes the protein required for adenovirus self-replication. RDAd-p53 includes Gendicine [24], Advexin [118], and SCH-58500 [119], all of which are recombinant type 5 adenoviruses (Ad5) containing *WTp53*. Gendicine was approved by the China Food and Drug Administration for the treatment of head and neck squamous cell carcinoma in 2003 and was successfully launched in 2004. Currently, it has accumulated a wealth of clinical application experience [24]. Gendicine has a large number of clinical use records in head and neck cancer [24], lung cancer [120], cervical cancer [121], ovarian cancer [122], uterine sarcoma [123], liver cancer [124], kidney cancer [125], pancreatic cancer [126], esophageal cancer [127], colorectal cancer [128], melanoma [21], and other tumor diseases, showing a certain safety and effectiveness. No serious adverse consequences related to Gendicine have been found yet. In addition, Gendicine can also be used to alleviate cancerous pleural and abdominal effusion, with the majority of patients using Gendicine experiencing a decrease in fluid volume, while only a small number of patients have no improvement or deterioration in their condition [129,130]. Adnexin has shown some effectiveness in clinical trials of many types of tumor diseases, but due to a lack of sufficient clinical trial data, Adnexin’s listing application was withdrawn in 2008 [118,131]. SCH-58500 has been clinically tested in ovarian cancer, but it has not been further developed thereafter [119].

CRAd-p53 includes ONYX-015, Oncoline, OBP-702, SG600-p53, etc. They are also known as oncolytic viruses. They can not only specifically express p53 in tumor cells, but also cleave cells through extensive replication and release tumor antigens and immune stimuli to induce anti-tumor activity [132]. The conditional replication mechanism of ONYX-015 and Oncoline is similar, both achieved by constructing *E1B-55K*-deficient adenoviruses. This is because studies have found that the protein encoded by the *E1B* gene of adenoviruses can inactivate p53, while adenoviruses with *E1B* gene deficiency can selectively replicate and lyse in p53-deficient human tumor cells, killing tumor cells, but they do not replicate and lyse in normal p53 cells [133]. ONYX-015 has also undergone a series of clinical trials, but the efficacy is not ideal in some trials [134]. Moreover, studies have found that ONYX-015 can replicate independently of the p53 state in cells; thus, its tumor specificity is controversial [135]. Oncoline was approved by China National Medical Products Administration for the treatment of nasopharyngeal carcinoma in 2005, but it subsequently experienced a period of stagnation. It was not until recently that people began to increase clinical research on Oncoline. A series of clinical studies on Oncoline have been conducted in cervical cancer [136], liver cancer [137], colorectal cancer [138], malignant pleural effusion, and ascites [139], and the current results show that the therapeutic effect is ideal. In OBP-702, the expression of adenovirus *E1A* gene is driven by the human telomerase reverse transcriptase (hTERT) promoter, and *WTp53* is inserted into the *E3* region of the adenovirus. Due to the high activity of telomerase in cancer cells and its quiescent state in most normal somatic cells, hTERT is the catalytic subunit of telomerase. Therefore, OBP-702 claims to have the ability to specifically infect and lyse tumor cells, Currently, OBP-702 has been proven to have significant anti-tumor effects in in vitro experiments [29]. OBP-702 is developed from oncolytic adenovirus OBP-301, which has entered the clinical trial stage with good tolerance and efficacy. Therefore, OBP-702 also has great clinical development potential [140]. SG600-p53 is an Ad5 that lacks the CR2 region of the *E1A* gene. The expression of the *E1A* gene in SG600-p53 is driven by the hTERT promoter, the expression of the *E1B* gene is controlled by the hypoxia-responsive element, and the *p53* gene is controlled by the cytomegalovirus promoter. Currently, SG600-p53 exhibits selective tumor killing ability in vitro [30].

The major drawback of adenoviruses is their strong immunogenic effects, often leading to side effects such as fever in patients taking rAd-p53. Fortunately, these fever varieties usually have self-limiting effects. However, the prevalent presence of Ad5 neutralizing antibodies across various groups might weaken the efficacy of rAd-p53, possibly limiting its usage [141,142]. The coxsackie-adenovirus receptor facilitates the adherence of adenoviruses to cellular surfaces, with their internalization facilitated through interactions with integrins belonging to the αvβ3 and αvβ5 categories [143]. The presence of integrins on certain tumor cell surfaces can significantly boost the capacity for adenovirus-specific tumor infections [144]. Nonetheless, the scant presence of coxsackie-adenovirus receptors on the exterior of cancerous cells like ovarian cancer could impede the use of rAd-p53 in these cases [143]. To overcome this problem, researchers have developed new tumor receptors other than CAR and introduced elements that can specifically recognize these receptors into adenovirus, thus constructing a novel recombinant adenovirus to attempt to promote adenovirus infection of tumor cells, which is beneficial for adenovirus to be applicable to more types of cancer to some extent [145].

#### 3.1.3. Vaccinia Virus Vectors

Vaccinia virus vectors does not integrate the viral genome into the host genome and has been used as a live vaccine against smallpox. In the field of vaccines, a modified vaccinia virus Ankara containing the *WTp53* gene is undergoing clinical trials and has shown some positive effects in anti-tumor treatment [146]. Nonetheless, the use of vaccinia virus vectors in gene therapy remains uncommon. Early on, Fodor et al. [34] successfully mediated *p53* gene therapy using vaccinia virus vectors and found that it prolonged the average survival rate of mice carrying tumors. However, the recombinant vaccinia virus p53 has not been put into the next clinical trial, and its safety still needs further experimental data confirmation. In addition, thymidine tyrosine kinase-deficient cowpox virus has been shown to selectively infect tumor tissues, while the virus’s infectivity and replicability in normal cells are weakened [147], providing an effective delivery method for anti-tumor gene therapy.

#### 3.1.4. Phage Vector

While the previously referenced viral vectors excel in gene transduction, their inherent affinity for human cells allows for the virus to invade and harm regular cells. Consequently, attaining accurate gene transfer to the tumor location presents a challenge for them. While localized administration like intratumoral injections can partially prevent medication harm to the usual area, precise delivery proves challenging in the context of tumors or non-solid tumors with extensive metastatic scope. Recently, bacteriophages have also been used as vectors in research on gene therapy [148,149]. In contrast to mammalian viruses, bacteriophages lack an innate inclination towards mammalian cells and tissues, thus possessing the capability to selectively transport genes to tumors via alteration and modification. Jordi et al. [97] prepared a tumor-specific RGD4C phage vector by expressing the tumor targeting ligand RGD on the M13 phage capsid. They claimed that the vector has the potential to allow for systemic delivery and demonstrated through experiments that it can efficiently deliver CRISPR-Cas9-*p53* gRNA to human lung cancer cells, successfully knocking out p53 protein expression. The phage vector is capable of enduring a prolonged period at 4 °C, facilitating its storage and transportation. However, the process by which bacteriophage genomes are expressed in eukaryotic cells remains ambiguous, and the existence of possible safety hazards is yet to be verified.

### 3.2. Non-Viral Vector Mediated p53-Based Gene Therapy

Scientists have developed a range of non-viral genetic vectors utilizing cationic liposomes, cationic polymers, inorganic substances, polymer carbon nitride (PCN), exosomes, ultrasound microbubbles, and bacteria, utilizing them in *p53* gene therapy. Typically found at the nanoscale, these non-viral vectors take forms like nanospheres [150], nanosheets [45], nanotubes [151], nanorods [152], and core-shell configurations [16]. Compared with viral vectors, non-viral vectors are characterized by lower immunogenicity, high gene loading, easy modification and functionalization, low cost of preparation, and have the potential for mass production and application. Nonetheless, it is common for non-viral vectors to exhibit low efficiency in gene transfection.

#### 3.2.1. Cationic Liposomes

Cationic liposomes excel in gene transfection and biocompatibility, positioning them as the quickest evolving non-viral vectors. A liposome nanocomposite modified with anti-transferrin receptor single chain antibody, named SGT-53, has completed phase I clinical trials in patients with advanced solid tumors [153]. SGT-53 can specifically recognize transferrin receptors overexpressed on the surface of tumor cells, thereby improving the accuracy of tumor treatment. Most patients who use SGT-53 have stable or improved conditions, and no serious adverse reactions have been observed [153]. At present, SGT-53 has entered the phase II clinical trial stage and has achieved good mid-term results [35].

#### 3.2.2. Cationic Polymers

Polyethyleneimine (PEI) [36], polyamide amine dendritic polymer (PAMAM) [37,154], chitosan [155], oligoethylimine [38], poly (β-aminoester) [39], poly (2-dimethylamino) ethyl methacrylate [40,41], and some cationic peptides have all been applied in research related to *p53* gene therapy. Similar to various cationic substances, cationic polymers have the ability to attach to negatively charged nucleic acid medications via electrostatic forces. A dense positive charge on the carrier’s surface can amplify its engagement with the anionic proteoglycans on the target cell’s surface, thus facilitating the penetration of nucleic acid medications into cells. Nonetheless, intense electrostatic forces have the potential to harm the cell membrane and mitochondria in regular cells. Consequently, a notable paradox exists between the efficiency of transfection and the cytotoxic effects of numerous cationic carriers.

PEI and PAMAM have a large number of amine groups in their structures, and carriers rich in secondary and tertiary amine groups can become “proton sponges” with strong proton buffering ability. When entering lysosomes, they absorb a large amount of hydrogen ions, causing chloride ion influx, resulting in lysosomal rupture and the release of nucleic acid drugs [156]. Therefore, PEI and PAMAM have excellent lysosomal escape ability and high gene transfection efficiency but often also high cytotoxicity. Chitosan, oligoethylimine, and other materials generally have good biocompatibility and biodegradability and low cytotoxicity, but they often suffer from low transfection efficiency. Therefore, balancing the relationship between transfection efficiency and cytotoxicity is crucial for the advancement of cationic polymers. At present, many new composite carriers based on cationic polymerization have been derived [42,71,157,158]. These new carriers have been modified, and their shortcomings have been improved to a certain extent, but they have not yet demonstrated clinical applicability.

Some cationic peptides or positively charged proteins have also been used as gene carriers in research on *p53* gene therapy, such as trans activator protein (TAT) [159], polyarginine peptides [160], polylysine [161], histones [17], etc. TAT and polyarginine peptides have strong cell penetrability, but they are easily recognized by the reticuloendothelial system during gene delivery to cells and thus are cleared by the body. Therefore, they are generally only used to condense genes or modify delivery systems to improve cell penetrability and cannot be delivered alone. Polylysine has good biodegradability, but the gene transfection efficiency is not ideal. As a naturally positively charged protein, histones have the advantage of relatively good biocompatibility, but they often rely on other materials to achieve efficient gene delivery.

#### 3.2.3. Inorganic Nanocarriers

The surface positive charge density of inorganic nanocarriers is generally low, and their gene-loading capacity is poor. Therefore, they typically connect cationic polymers to enhance nucleic acid-loading capacity. Many inorganic nanocarriers, such as superparamagnetic iron oxide nanoparticles (SPIO-NPs) [162], gold nanoparticles [43], and silica nanoparticles [44], possess unique optical, electrical, and other physical properties, which endow them with imaging, targeted delivery, and other functions. However, there are also significant bottlenecks in the clinical development of these inorganic nanocarriers, and their metabolic pathways in the body are not clear. Moreover, many of these inorganic nanocarriers have poor biodegradability, which may cause certain damage to the body.

In addition, carbonate inorganic nanomaterials such as calcium carbonate nanoparticles [150] and carbonate apatite nanoparticles [163] have also been used in basic research of *p53* gene therapy. They have advantages such as excellent biocompatibility and low toxicity. Carbonate apatite nanoparticles are pH sensitive and can specifically cleave and release nucleic acid drugs in tumor environments with relatively low pH, but they have not shown the feasibility of clinical application.

#### 3.2.4. PCN

PCN is a graphene-like material with high intensity photoluminescence intensity and large specific surface area [45]. Mingxuan et al. [45] constructed a photo-responsive non-cationic *p53* gene delivery system based on highly water-dispersible PCN nanosheets. The PCN nanosheets overcame the macroscopic size, poor water dispersibility, and poor photo-responsiveness of traditional block PCNs, exhibiting excellent lysosomal escape ability. Compared with cationic carriers, they also avoided cytotoxicity caused by high cationic charge units.

#### 3.2.5. Exosomes

Exosomes are extracellular vesicles released by cells, with lipid bilayer membranes [164]. Exosomes as drug delivery carriers have advantages such as a long cycle half-life and excellent biocompatibility. Radha et al. [46] combined exosomes isolated from colostrum powder with PEI to form a novel gene delivery system EPM, and demonstrated that the exogenous *p53* plasmid delivered by EPM can be expressed in lung tumors and mouse cell tissues, and EPM exhibits low cytotoxicity and good biocompatibility.

#### 3.2.6. Microbubbles

Luca et al. [165] used microbubbles as gene delivery vectors, combining them with ultrasound to induce p53 expression in liver cancer cells. These microbubbles consist of sulfur hexafluoride encased in a phospholipid membrane. This combination allows for controlled drug release and targeted delivery of genetic therapies; however, ultrasound can potentially cause mechanical damage to normal tissues or cells.

#### 3.2.7. Bacteria Vector

Due to the relatively hypoxic tumor microenvironment (TME), certain facultative and specialized anaerobic bacteria are believed to have a strong tendency to colonize tumors [166]. Genesy et al. [47] utilized a highly attenuated strain of *Salmonella typhimurium* (χ 11218) to deliver the *p53* gene to human bladder cancer cells in vitro. This strain can effectively transport genes to the cytosol of target cells through its bacterial expression system, thereby inducing apoptosis in bladder cancer cells. While bacterial vectors present a cost-effective option, further in vitro and in vivo studies are needed to confirm the safety of this delivery method.

### 3.3. Improvement of Non-Viral Vectors

It is a research hotspot to improve the function of non-virus vectors and develop new multifunctional delivery systems. The inefficiency of non-viral vectors in gene transfection is associated with their role in targeting tumors, entering cell membranes, and evading lysosomes. Academics have extensively endeavored to enhance these capabilities, a topic we will delve into subsequently.

#### 3.3.1. Improve the Vector’s Capacity to Specifically Target Tumors

**Figure 4:**
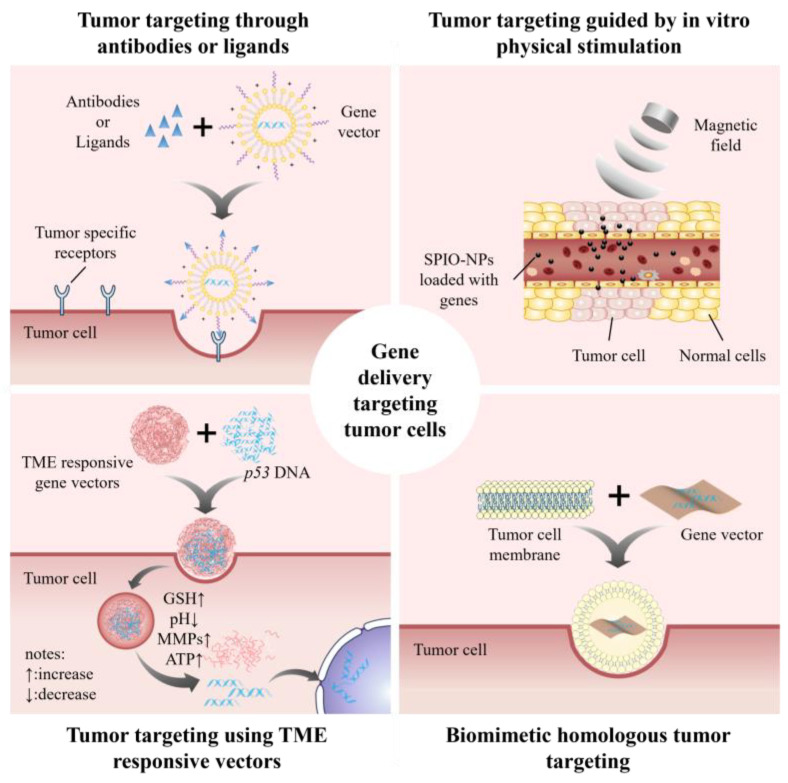
Methods to improve the vector’s capacity to specifically target tumors.

Regarding the synthesis of nucleic acid drugs, the accuracy of gene therapy targeting tumors can be improved by incorporating promoters specific to tumors. In addition, several methods can be used to improve the tumor-specific targeting function of vectors, including modifying antibodies or ligands on the surface of vectors, preparing vectors that respond to the TME, preparing vectors that respond to physical stimuli in vitro, and using biomimetic homologous materials (illustrated in Figure 4). Targeting tumors specifically with carriers can lessen the harm nucleic acid medications cause to healthy cells, decrease drug misuse, and enhance the precision of tumor therapy.

Tumor targeting through antibodies or ligands are described as follows: By attaching corresponding ligands or antibodies to the surface of the vector, it is possible to target highly expressed receptors or antigens in tumor cells or neovascular endothelial cells. Ligand properties include peptides, vitamins, polysaccharides, etc. Peptide ligands are characterized by easy screening and synthesis and good specificity but high immunogenicity, such as antibodies. Vitamin and polysaccharide ligands usually have good biocompatibility and weak immunogenicity but lack effective screening methods. Scientists have created a range of *p53* gene treatments aimed at different cancer-specific receptors, such as epidermal growth factor receptors [167], folate receptors [48], CD44 receptors [44,152], transferrin receptors [153], neurociliary protein [71], asialoglycoprotein receptors [161], and integrin receptors [97]. However, many of the aforementioned tumor receptors are also mildly expressed on the surface of normal cells, resulting in poor tumor-specific efficacy. Consequently, there remains a need to explore tumor targets that are more representative or universally applicable. Researchers also directly screened ligands with high affinity for tumor cells as receptors. For example, Sukuma et al. [73] used phage display random peptide technology to screen high-affinity ligand SP94 peptide for HCC (liver cancer cell line) and modified the *p53* gene delivery system with this SP94 peptide to achieve a targeted delivery of gene drugs. The peptide ligands screened by phage display random peptide technology have a small volume and do not affect the size of the carrier or gene delivery efficiency. The diverse range of tumor cell mutations can lead to certain diseases struggling to locate appropriate targeted medications. Consequently, there is an immediate need to extensively employ current affinity ligand screening methods to diversify the range of targeted vectors or medications.

Tumor targeting using TME-responsive vectors are described as follows: Compared with normal tissue, the TME has characteristics such as relative hypoxia, high H_2_O_2_ content, high GSH concentration, low pH, an overexpression of tumor-related enzymes, and high levels of ATP [168]. Therefore, TME-sensitive chemical bonds or macromolecules were introduced into vectors to construct TME-responsive delivery systems that can specifically release drugs in tumor cells. Some typical TME-responsive vectors in *p53* gene therapy are shown in Table 2.

Targeting guided by in vitro physical stimulation is described as follows: The introduction of substances sensitive to physical stimuli, including SPIO-NPs [162], photosensitizers [45], etc., into the vectors, which are then guided through in vitro physical stimuli (magnetism, optics, etc.), enables greater drug release to tumor cells. SPIO-NPs are magnetically responsive and can be directed to a tumor site with external magnetic fields [162]. The build-up of ROS from photosensitizers when exposed to light can interfere with lysosome structures, thus facilitating the release of *p53* genes from lysosomes [45]. After introducing the photosensitizer into the vector, the drug release of the target can be triggered by controlling external light irradiation.

Biomimetic homologous tumor targeting is described as follows: This method involves wrapping tumor cell membranes around the surface of a vector, disguised as endogenous material, to deliver drugs against homologous tumor cells. For example, Zhou et al. [36] coated the surface of a *p53* gene vector with B16F10 cell membrane, and the results showed that the vector has a highly specific targeting effect on homologous cancer cells.

#### 3.3.2. Improve the Vector’s Capacity to Penetrate the Cell Membrane

In cases where non-viral vectors struggle to infiltrate cell membranes, it is common for scientists to affix protamine [48], TAT [158,171,172], peptides abundant in arginine [49], or peptides high in lysine [71] to the vector’s exterior. The surface of these materials is abundant in positive charges, enhancing the bond between the carrier and the cell membrane, thus facilitating the carrier’s penetration into the cell.

#### 3.3.3. Improve the Vector’s Capacity to Escape Lysosomes

The capacity of the vector to elude lysosomes may negate the decomposition of nucleic acid drugs by lysosomes. The currently discovered methods to further enhance the lysosomal escape ability of *p53* gene delivery vectors include modifying the vector with substances rich in histidine [159], modifying the vector with substances rich in amine groups [156], and combining *p53* gene therapy with photochemical therapy [45]. Substances rich in histidine undergo protonation in low pH environments of tumor cells, thereby promoting lysosomal escape of drugs. The mechanisms of the latter two have been mentioned earlier.

#### 3.3.4. Diminish the Vector’s Toxic Effects

The high cytotoxicity of cationic vectors is one of the reasons limiting their clinical application. Currently, the main method to reduce the toxicity of cationic vectors is to attach materials with charge-shielding properties to the carrier, such as polyethylene glycol [50,73], hyaluronic acid [152], etc. However, charge-shielding materials may lead to a decrease in the efficiency of the vector’s membrane entry. Therefore, a tumor-environment-sensitive chemical bond is also needed to connect the charge-shielding material with the vector. When vectors reach tumor cells, chemical bonds degrade, and vectors remove charge barrier substances, allowing for more efficient entry into tumor cells.

The previously mentioned methods have somewhat improved the efficiency of gene transfection using non-viral vectors. Nonetheless, numerous critical concerns remain: Initially, the inability of the body to break down the modifier, leading to its substantial accumulation in regular cells, could result in possible toxic impacts on the body. Consequently, it is essential for the modifier to maintain high biocompatibility and biodegradability while reducing harmful side effects on the body to the greatest extent. Additionally, the modifier’s dimensions must be suitable to prevent obstructing drug entry into the cell membrane or nucleus for their impact. As a third point, the modification of modifiers on the carrier typically occurs via methods of physical embedding or chemical bond coupling. Should the chemical bonds remain undegraded or possess low biodegradability within the body, it could lead to bodily harm. Consequently, it is essential for chemical bonds to possess high biodegradability. Furthermore, the alteration technique must maintain the carrier’s steadiness throughout the delivery phase to prevent the modifier from detaching prior to arriving at the intended location.

### 3.4. Multiple Drugs Co-Delivery System

In the basic research of combination therapy, researchers have created a series of multiple-drug co-delivery systems to lessen drug administration frequency, ease medical staff workload, and lessen patient medication load. Cationic liposomes [173], mesoporous silica nanoparticles [174,175], pullulan derivatives [176], cyclodextrin (CD) [177], among others, serve as carriers in the development of co-delivery systems for the *p53* gene and various other medications. The distinct physical and chemical characteristics of these carriers enable them to transport various drugs at once.

Possessing amphiphilicity, cationic liposomes are capable of loading *p53* genes and also encasing drugs that dissolve in water or lipids. Mesoporous silica nanoparticles possess an extensive specific surface area and pore dimensions and a robust ability to load drugs, and their surfaces are readily alterable, enabling their combination with cationic carriers for simultaneous gene and drug delivery. Pullulan is a kind of water-soluble polysaccharide with no electricity, is non-toxic and edible, and has excellent biocompatibility. Its structure is rich in hydroxyl groups, making it easy to modify. Modifications involving hydrophobic and cationic elements can be utilized on pullulan to create co-delivery systems for the *p53* gene and hydrophobic medications. CD has a hydrophilic surface and a hydrophobic inner cavity to encapsulate hydrophobic drugs. The combination of CD and gene carriers enables the synergistic delivery of genes and hydrophobic drugs.

Researchers have developed multiple-drug co-delivery systems featuring core-shell or double-walled microspheres to address the issue of rapid initial drug bursts, enhancing drug efficacy through synergistic sustained-release mechanisms. Pooya et al. [16] prepared core-shell particles using poly (lactic acid glycolic acid) and poly (D, L-lactic acid) as core and shell materials, respectively. The core layer was loaded with nutlin-3a, which can restore the apoptotic activity of p53 protein by disrupting the interaction between Mdm2 and p53. The shell chamber was loaded with chitosan and the *p53* gene complex. The results showed that drugs delivered using this core-shell structure have a good release curve and can sustainably release drugs at lower doses for a long time.

While co-delivery systems for genes and drugs can be more convenient in the medical field, the development and design of co-delivery systems face many challenges. First, the site of action, release sequence, and release time of different drugs may vary. For example, *p53* DNA typically targets the nucleus, while siRNA targets the cytoplasm. Therefore, a co-delivery system usually requires some controlled release function to release the drug at a specific time and location. Second, the synergistic drug delivery system is generally large and has difficulty crossing biological barriers, such as endothelial cells, during drug administration, affecting drug delivery efficiency.

## 4. Overview and Outlook

WTp53’s potent anti-cancer properties, coupled with early outcomes from *p53*-based gene therapy, have instilled renewed optimism in cancer sufferers. Initially, the aim behind creating *p53*-based gene therapy was to eradicate tumors from their origin, yet this ultimate objective remains elusive. The *WTp53* replacement therapy is extensively researched and utilized, yet it fails to rectify or eliminate abnormal genes, and its effectiveness is frequently deemed inadequate in clinical settings. While researchers have somewhat reduced the limitations of this treatment, including through a series of multidrug combination strategies, it still lacks unique features and advantages compared to other cancer treatments. Consequently, surmounting its weak anti-cancer properties or enhancing its unique capabilities is crucial for its continued advancement. Fisher M. in his article [178] noted that p53 has approximately 3661 target genes. This complexity complicates the assessment of whether excessive WTp53 expression could be harmful to the body, as the introduction of the *p53* gene may pose unknown risks in healthy cells. To mitigate potential toxic effects on normal cells, gene therapy must employ targeted gene delivery systems and localized administration to prevent the drugs from affecting healthy tissue. While the treatment involving the suppression or modification of the *MTp53* gene remains untested in clinical settings, in theory, it holds significant promise for clinical use. The primary limitation of this treatment lies in the possibility of deviating from the intended risk. Accidental alterations to other genes during therapy could lead to bodily harm. Therefore, it is crucial to continuously upgrade gene editing tools and establish sound risk prediction and safety assessment procedures.

Furthermore, we can focus directly on the downstream target genes of p53. Therapies based on these downstream targets, including the aforementioned miR-34a therapy, have shown promising potential. The *PUMA* gene is a downstream target of p53 that can induce cell apoptosis through both p53-dependent and p53-independent pathways. Recently, the development of *PUMA* gene therapy has shown effectiveness in reducing inflammation, and exploring its potential for tumor treatment is also promising [179].

The advancement of gene therapy heavily relies on the technology of delivering nucleic acids. The efficiency of viral vectors in gene delivery has made them widely popular. In particular, the extensive and prolonged use of adenovirus vectors in clinical settings highlights their relative safety and effectiveness. Moreover, the safety and efficacy of lentiviral vectors have steadily improved over the past few years. Known for their ability to support stable gene expression over extended periods, lentiviral vectors have emerged as highly promising options in clinical settings. However, concerns remain regarding their immunogenic properties and the high costs associated with viral vectors. Frequent use of these vectors can lead to the development of antiviral immunity in the body, weakening the effectiveness of the drug. In recent years, various non-viral vectors have been developed and put into use, offering a promising alternative to viral vectors. Among these, cationic liposomes stand out due to their favorable biocompatibility and effective gene transfection, making them one of the most widely used non-viral vectors today. A wide array of records documents the use of cationic liposomes in both laboratory settings and clinical disease management. They have demonstrated significant safety and effectiveness across various applications, underscoring their considerable potential for clinical development. However, many other non-viral vectors struggle to achieve a balance between gene transfection efficiency and biocompatibility. While numerous scientists are improving it via chemical alterations and various techniques, adding modifiers will elevate both safety hazards and financial expenses. Indeed, the primary function of vectors lies in accurately targeting tumors and administering nucleic acid medications, enabling gene therapy to inflict minimal damage on the human body and adjust to various tumor conditions, including metastatic or non-solid tumors. Despite numerous vectors asserting a precise delivery of nucleic acid medications to tumors via systemic delivery, their practical application in clinical settings remains pending. At present, gene therapy still has some limitations in indications and administration methods. Consequently, enhancing fundamental studies on the mechanisms of tumor diseases and the traits of TMEs, developing more representative targets for tumors, investigating substances sensitive to the TME, and identifying more secure and efficient carriers are crucial for advancing gene therapy.

## Figures and Tables

**Table 1:** Advancements in research on certain *p53* gene therapy products.

Name	Vector	Phase	Indication	Outcome	Ref.
Gendicine	RDAd	Approved in China	Head and neck squamous cell carcinoma, etc.	Safe and effective.	[24]
INGN 201 (Advexin)	RDAd	I/II	Esophageal squamous cell carcinoma, etc.	Safe, feasible, and biologically active.	[25]
SCH-58500 (ACN53)	RDAd	I/II	Recurrent ovarian cancer, primary peritoneal cancer, fallopian tube cancer, etc.	Safe and well-tolerated.	[26]
ONYX-015 (dl1520)	CRAd	I/II	Advanced sarcoma, etc.	Well-tolerated.	[27]
Oncoline (H101)	CRAd	Approved in China	Nasopharyngeal carcinoma, etc.	Safe and effective.	[28]
OBP-702	CRAd	In vitro and in vivo	Diffuse-type gastric cancer cells	Inhibit tumor growth in mice and induce cell apoptosis and autophagy.	[29]
SG600-p53	CRAd	In vitro and in vivo	Non-small cell lung cancer cells	Selective replication in tumors and anti-tumor effects.	[30]
SG635-p53	CRAd	In vitro and in vivo	Liver cancer cells	Inhibit tumor growth and prolong animal survival.	[31]
AdDelta24-p53	CRAd	In vitro and in vivo	Malignant glioma, etc.	Relieve tumors and prolong animal survival.	[32]
dl922-947	CRAd	In vitro and in vivo	Breast cancer cells	Demonstrate anti-tumor efficacy in vivo and in vitro.	[33]
rVV-TK-53	Vaccinia virus	In vitro and in vivo	Bladder cancer cells	Inducing the extinction of tumor cells.	[34]
SGT-53	Liposome nanoparticles	II	Pancreatic adenocarcinoma	Clinically significant benefits	[35]
CM@MnO_2_-PEI-NLS-ss/p53	Polyethylenimine	In vitro and in vivo	Malignant melanoma cells	Targeting cancer cells with high specificity and inhibiting tumor growth.	[36]
AP-PAMAM/p53	Polyamide amine dendritic polymer	In vitro	Cervical cancer cells	Anti-tumor proliferation, induction of cell apoptosis, inhibition of cancer cell migration and invasion.	[37]
LA-OEI/p53	Lipoic acid-modified oligoethylenimine	In vitro	Cervical cancer cells	Inhibit cell migration.	[38]
PBAE537	Poly (Beta-Amino Ester) Complex Nanoparticles	In vitro and in vivo	Cervical cancer cells	Successfully reversed cervical intraepithelial neoplasia in HPV transgenic mice.	[39]
DPD/pEGFP-C1-p53	Dextran-graft-poly ((2-dimethyl amino) ethyl methacrylate)	In vitro and in vivo	Breast cancer cells	Inhibit tumor cell proliferation.	[40]
micelles/DOX/p53	POSS-based star-shaped polymer	In vitro and in vivo	Breast cancer cells	Inducing apoptosis of tumor cells.	[41]
P-CSSO/p53	PEG modified glycolipid-like polymer	In vitro and in vivo	Liver cancer cells	The tumor inhibition rate can reach 77.1%.	[42]
AuNPs-p53	Gold nanoparticles	In vitro	Lung cancer cells	Low toxicity in normal cells, triggering apoptosis in tumor cells.	[43]
MB-NSi-p53-CS	Silica-polymer composite nano system	In vitro and in vivo	Lung cancer cells	Low cytotoxicity, high *p53* transfection, and anticancer efficacy.	[44]
PCN-P53	Highly water-dispersible polymeric carbon nitride (PCN) nanosheets	In vitro and in vivo	Cervical cancer cells	Efficient DNA condensation, outstanding biocompatibility, transfection tracking, light responsiveness, and high transfection efficiency.	[45]
EPM-pcDNA-p53	Bovine colostrum exosomes and polyethyleneimine matrix	In vitro and in vivo	Lung cancer cells	Inhibiting the proliferation of tumor cells.	[46]
χ11218 pYA4545p53 strain	χ11218 strain of *Salmonella typhimurium*	In vitro	Bladder cancer cells	Decrease the vitality of human bladder cancer cells.	[47]
p53/C-rNC/L-FA	Liposome nanoparticles	In vitro and in vivo	Breast cancer cells	Inducing tumor cell apoptosis and inhibiting tumor growth.	[48]
RHD/p53	Cationic peptide	In vitro and in vivo	Cervical cancer cells	Demonstrate anti-tumor efficacy in vivo and in vitro.	[49]
FK/p53/PEG-PLL (DA)	Cationic peptide	In vitro and in vivo	Cervical cancer cells	Demonstrate anti-tumor efficacy in vivo and in vitro.	[50]

Note: RDAd: replication defective adenovirus; CRAd: conditional replication adenovirus.

**Table 2:** TME-responsive vectors in *p53* gene therapy.

TME-Responsive Vectors	TME-Sensitive Chemical Bonds or Macromolecules	Targeting Mechanism	Ref.
Redox-responsive vector	Disulfide bond	The high content of GSH in tumor cells can trigger disulfide bond cleavage, thereby releasing drug complexes within tumor cells.	[49]
PH-responsive vector	Hydrazone	The low pH of tumor cells can promote pH-sensitive chemical bond cleavage, thereby releasing drug complexes within tumor cells.	[49]
Enzyme-responsive vector	CPLGIAG peptide	Matrix metalloproteinases (MMPs) are overexpressed in almost all human tumors, and CPLGIAG peptides can be hydrolyzed by MMPs. The drug is coupled to the vector through CPLGIAG peptides, and after entering tumor cells, CPLGIAG peptides are hydrolyzed by MMPs to achieve targeted drug release.	[71]
	Phosphoester bond	The content of phosphodiesterase I in tumor cells is higher than that in normal tissues. Phosphate ester bonds are degraded by phosphodiesterase, and polyphosphate esters rich in phosphate ester bonds are used as *p53* gene vectors. After entering tumor cells, the phosphate ester bonds are degraded to release drugs.	[169]
ATP-responsive vector	ATP-responsive aptamer duplex	The level of ATP in the intracellular fluid is higher than that in the extracellular environment. In the ATP rich tumor environment, the structural changes of ATP-responsive aptamer duplex release, thereby targeting the release of loaded drugs.	[170]

## Data Availability

Not applicable.
